# Tumor Mutational Burden and Clinical Outcomes in Patients with Muscle-Invasive Bladder Cancer Treated with Neoadjuvant Chemotherapy

**DOI:** 10.1158/2767-9764.CRC-26-0255

**Published:** 2026-08-27

**Authors:** Earle F. Burgess, Landon C. Brown, Michael McCormack, Liang Liu, Nury Steuerwald, Ericson Stoen, Metamia Ciampricotti, Patrick M. Doherty, Edward Williams, Peter E. Clark, Wei Zhang, James T. Symanowski

**Affiliations:** 1 Atrium Health Wake Forest Baptist Comprehensive Cancer Center, Charlotte, North Carolina.; 2 Atrium Health Wake Forest Baptist Comprehensive Cancer Center, Winston-Salem, North Carolina.; 3 Tempus AI, Inc., Chicago, Illinois.

## Abstract

Pathologic complete response (pCR) after cisplatin-based neoadjuvant chemotherapy (NAC) is associated with improved outcomes in muscle-invasive bladder cancer (MIBC), but genomic predictors of benefit remain unvalidated. Based on observations in lung cancer, we hypothesized that patients with MIBC with high tumor mutational burden (TMB) would be unlikely to benefit from NAC, indicated by residual disease after cystectomy. Pretreatment tumor specimens from patients treated with cisplatin-based NAC were analyzed using Tempus xT/xR platforms. Sample size was prespecified by statistical design. High TMB was defined as the upper TMB quintile within the cohort. Correlation of TMB values and genomic variants with outcomes was performed using logistic regression, Cox proportional hazards models, and Kaplan–Meier techniques. Ninety-one patients were included, with a median follow-up of 63.6 months. pCR occurred in 31.9%. Median TMB was 8.9 mutations per megabase (Mut/Mb). Contrary to the prespecified hypothesis, higher TMB was associated with more favorable outcomes. Patients in the upper TMB quintile had a numerically higher pCR rate (47.4% vs. 27.8%; *P* = 0.165) and improved relapse-free survival (RFS)/overall survival [OS; hazard ratio (HR), 0.456; *P* = 0.08 and HR, 0.495; *P* = 0.16]. Using a clinically relevant cutoff, TMB ≥ 10 Mut/Mb was associated with higher pCR [43.6% vs. 23.1%; odds ratio (OR), 0.39; *P* = 0.044] and improved RFS (HR, 0.330; *P* < 0.001) and OS (HR, 0.388; *P* = 0.013). Exploratory analyses showed a trend toward enrichment of mismatch repair alterations (OR, 3.57; *P* = 0.087) in TMB-high tumors. Baseline TMB ≥ 10 Mut/Mb was associated with improved pathologic response and survival in MIBC treated with cisplatin-based NAC and warrants prospective evaluation in contemporary perioperative regimens.

**Significance:**

In MIBC treated with cisplatin-based neoadjuvant chemotherapy, we found that baseline TMB ≥10 Mut/Mb was associated with higher pCR rates and improved RFS and OS. These findings support the prospective study of TMB as a prognostic biomarker in contemporary perioperative treatment regimens.

## Introduction

Despite recent therapeutic advances, patients with muscle-invasive bladder cancer (MIBC) have a poor prognosis and a significant chance of relapse with contemporary perioperative systemic regimens (1, 2). For those who are eligible, cisplatin-based neoadjuvant chemotherapy prior to radical cystectomy (RC) has been a standard treatment option and is associated with a modest reduction in the risk of death (3). More recently, the addition of perioperative immune checkpoint inhibition has shown improved outcomes compared with chemotherapy alone (1, 2).

Prior clinical studies have suggested that the presence of a pathologic complete response (pCR) after neoadjuvant chemotherapy (NAC) serves as a surrogate marker for improved overall survival (OS) in MIBC (4). Efforts to find genomic aberrations associated with pCR after NAC identified pathogenic mutations in candidate genes, including *ERBB2*, and DNA-repair enzymes, such as *ATM* and *ERCC2*, but these findings have not been prospectively validated nor consistently observed (5–8).

Intratumoral genomic heterogeneity underlies chemotherapy resistance through treatment-induced Darwinian selection of drug-resistant subclones, resulting in incomplete tumor responses and treatment failure (9–11). Intratumoral heterogeneity is also positively associated with tumor mutational burden (TMB; refs. 12, 13). High TMB increases tumor neoantigen load, which improves response to immune checkpoint inhibitors (ICI), but the impact of TMB on chemotherapy response is not well defined (14). In the post-NAC setting, increased genomic heterogeneity is associated with poor survival in MIBC (15). This observation suggests that high TMB in the treatment-naïve setting could be a surrogate for genomic heterogeneity and thus the probability of chemo-resistant subclones. This hypothesis is supported by retrospective studies in patients with lung cancer that found tumors with high TMB may have inferior responses to chemotherapy and survival (16–19).

TMB ≥ 10 mutations per megabase (Mut/Mb) predicts which patients are more likely to benefit from ICIs, but a specific TMB threshold prognostic for outcome or predictive for response to chemotherapy in MIBC is unknown. Based on the observed relationship between TMB and poor chemotherapy response in lung cancer, we designed this study to test the hypothesis that high baseline TMB identifies patients with MIBC who are unlikely to benefit from cisplatin-based NAC, defined by the presence of pathologic residual disease after RC. Additionally, genomic sequencing data were used for exploratory analyses to identify molecular variants associated with high TMB.

## Materials and Methods

### Study population

Chemotherapy-naïve transurethral resection of bladder tumor (TURBT) specimens were obtained from patients with MIBC treated with NAC followed by RC between January 1, 2009, and June 30, 2023, within the Atrium Health Wake Forest Baptist Comprehensive Cancer Center network. Eligible patients were ≥18 years of age with histologically confirmed muscle-invasive urothelial carcinoma of the bladder and available archival pretreatment tumor tissue. All patients received either neoadjuvant gemcitabine and cisplatin (G/C) or standard or dose-dense methotrexate, vinblastine, doxorubicin, and cisplatin (MVAC). Clinical data were abstracted retrospectively from institutional databases and electronic medical records. Data were stored in a secured, HIPAA-compliant limited dataset. This study was conducted in accordance with the Declaration of Helsinki and Good Clinical Practice guidelines. Waivers of informed consent and HIPAA authorization were granted due to the retrospective nature of the study and the use of archival specimens. The protocol (LCI-GU-MIBC-001R) was approved by the Atrium Health Institutional Review Board.

### Statistical analysis and design

The primary objective of the study was to assess the impact of high TMB on pathologic response rate in patients with MIBC treated with NAC and to test the hypothesis that high TMB, defined as the upper quintile (≥20%) of the cohort, was associated with an increased likelihood of residual disease after NAC. Secondary objectives included the correlation of TMB with relapse rates, relapse-free survival (RFS), and OS by quintile, tertile, and a threshold of 10 (Mut/Mb). Exploratory objectives included the analysis of gene variants and expression patterns associated with the upper TMB quintile.

Based on historical observations of a pathologic residual disease rate of 0.65 in all patients (4), the study was powered based on an assumed pathologic residual disease rate of 0.57 and 0.95 in the TMB nonhigh and high cohorts, respectively. With these prevalence assumptions and using a two-sided alpha of 0.05, a cohort size of 83 evaluable subjects was required to provide at least 90% power to detect this treatment effect size. Because genomic sequencing and specimen quality validation were conducted in sequential batches with a lag between processing and eligibility confirmation, additional cases were accrued before earlier samples were fully adjudicated, resulting in enrollment beyond the prespecified target. A total of 105 patients were evaluated, and adequate tumor material was present for sequencing in 91.

The primary analysis utilized logistic regression to model the probability of pathologic residual disease. An unadjusted odds ratio (OR) was estimated for high versus nonhigh TMB. Additional univariate logistic regression models were used to evaluate the prognostic significance of baseline clinical characteristics. Time-to-event outcomes were analyzed using Kaplan–Meier survival analyses and statistically tested using log-rank tests. Cox proportional hazards models were used to estimate hazard ratios (HR) for TMB and other covariates.

All statistical analyses were performed by biostatistics faculty at Atrium Health Wake Forest Baptist Comprehensive Cancer Center.

### Genomic analysis

Next-generation sequencing (NGS) of TURBT specimens was conducted using the Tempus xT assay (Tempus AI, Inc.) for the determination of TMB levels and identification of genomic variants as previously described (20, 21). Tempus xT is a targeted, tumor-normal–matched DNA panel that detects single-nucleotide variants, insertions and/or deletions, and copy-number variants in 648 genes, as well as chromosomal rearrangements in 22 genes with high sensitivity and specificity.

### Gene expression analysis

For specimens with adequate tumor material, RNA sequencing was also performed with the Tempus xR assay (Tempus AI, Inc.; refs. 22, 23). Tempus xR is a whole-transcriptome NGS assay spanning a 39 Mb target region of the human genome that quantifies transcript- and gene-level expression and identifies transcriptional evidence of chromosomal rearrangements resulting in the expression of fusion RNA species. Patients were grouped based on TMB status (upper quintile vs. the lower four quintiles). Salmon was used in quasi-mapping mode to provide high-accuracy transcript quantification while accounting for sequence-specific, fragment GC content, and positional biases with the human genome GRCh37 as a reference. Transcript-level quantifications were imported into the R statistical environment (v4.4.3) using the tximport package. Differential gene expression was performed using the DESeq2 package. Genes were considered significant if the adjusted *P* value (Benjamini–Hochberg) was less than 0.1, and the absolute log_2_ fold change was greater than 1.

## Results

### Patient characteristics and clinical outcomes

Ninety-one patients underwent NGS of chemotherapy-naïve TURBT tumor samples. Most patients were white (88%) and male (69%). The median follow-up for this cohort was 63.6 months (0.8–147.6 months). All patients received NAC prior to RC, which included G/C (64.8%) or MVAC (35.2%). Four (4.4%) patients received an adjuvant immune checkpoint inhibitor (ICI; Supplementary Table S1). A pCR was achieved in 29 (31.9%) patients.

**Figure 1.**
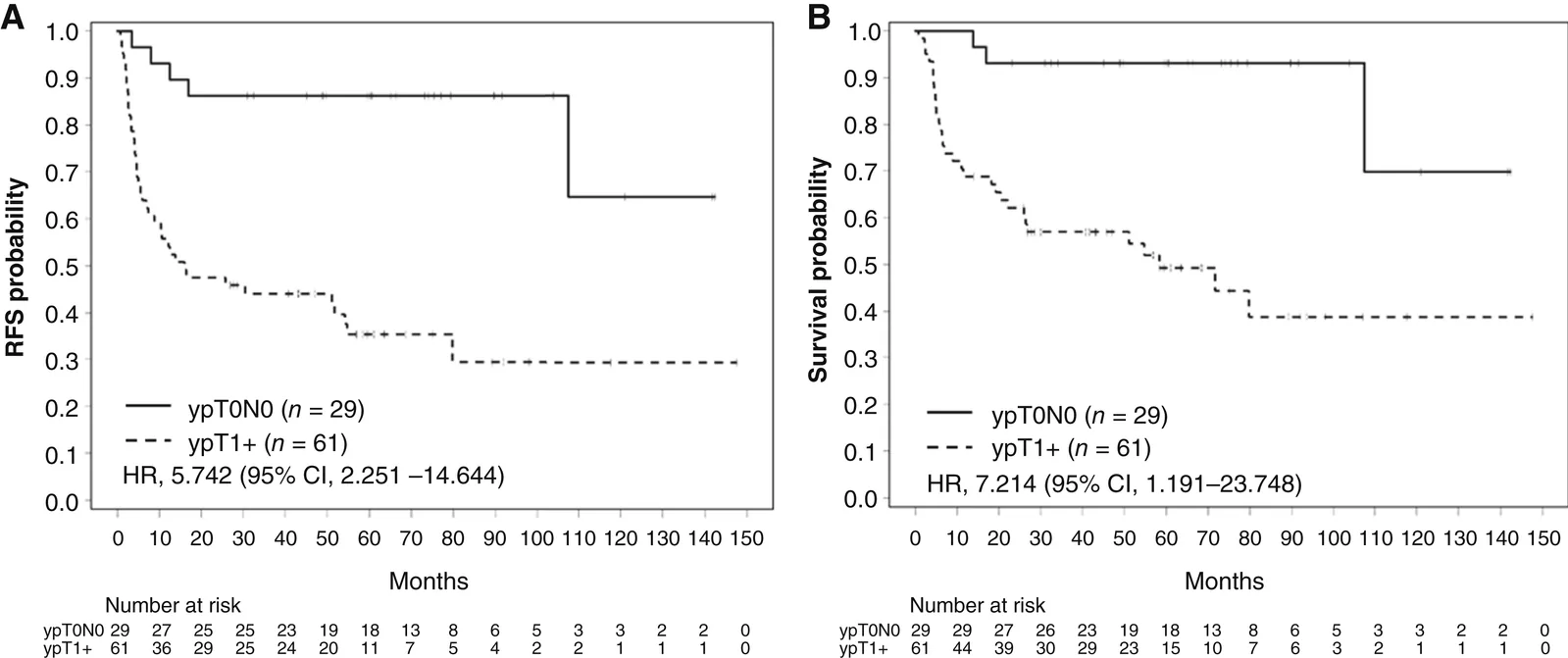
Survival outcomes by pathologic stage. **A,** RFS. **B,** OS. CI, confidence interval.

Thirty-eight (41.8%) patients relapsed, with a median time to relapse of 4.9 (IQR: 3–12.2) months. Patients who achieved a pCR had a very low relapse rate compared with those with pathologic stage I or greater disease (OR, 0.08; *P* < 0.001). No significant difference in relapse rate by chemotherapy regimen was observed (*P* = 0.182). The 5- and 10-year RFS rates were 52.4% and 40.7%, respectively, whereas the 5- and 10-year OS rates were 64.1% and 48.2%, respectively (Supplementary Fig. S1). For patients with pathologic stage I or greater, RFS [HR, 5.742 (2.251–14.644); *P* < 0.001] and OS [HR, 7.214 (1.191–23.748); *P* < 0.001] were significantly worse compared with those who achieved a pCR (Fig. 1).

### Association between tumor mutational burden and pathologic response

TMB (Mut/Mb) was calculated for all tumor specimens. The median TMB for the entire cohort was 8.9 (range, 1.1–27.9). The TMB threshold defining the upper quintile was 14.7. The TMB thresholds defining the upper and lower tertiles were 11.6 and 7.9, respectively. Thirty-nine (42.9%) tumors had a TMB of 10 or higher. The median TMB for patients who achieved a pCR was 11.1 (range, 3.7–27.4). For patients with pathologic stage I–IV (ypT1 or greater), the median TMB was 8.9 (range, 1.1–27.9).

The study was powered to assess the impact of the highest TMB quintile on pCR rates. Patients whose tumors had a TMB in the upper quintile achieved a pCR in 47.4% compared with 27.8% of patients with a TMB in the lower four quintiles (OR, 0.43; *P* = 0.165), a finding contrary to our initial hypothesis. Clinicopathologic features by TMB group are shown in Supplementary Table S1. We next analyzed pCR rates by TMB tertiles to determine if a continuous relationship between TMB level and pCR rate would be suggested. pCR rates occurred in 45.2% and 27.6% of patients with tumors in the upper and lower TMB tertiles, respectively (OR, 0.46; *P* = 0.138). As TMB ≥ 10 is associated with improved outcomes with the use of ICIs, we also examined the impact of this TMB threshold. Patients with a TMB ≥ 10 achieved a pCR in 43.6% compared with 23.1% with TMB less than 10 (OR, 0.39; *P* = 0.044).

### Association among TMB, relapse, and survival

**Figure 2.**
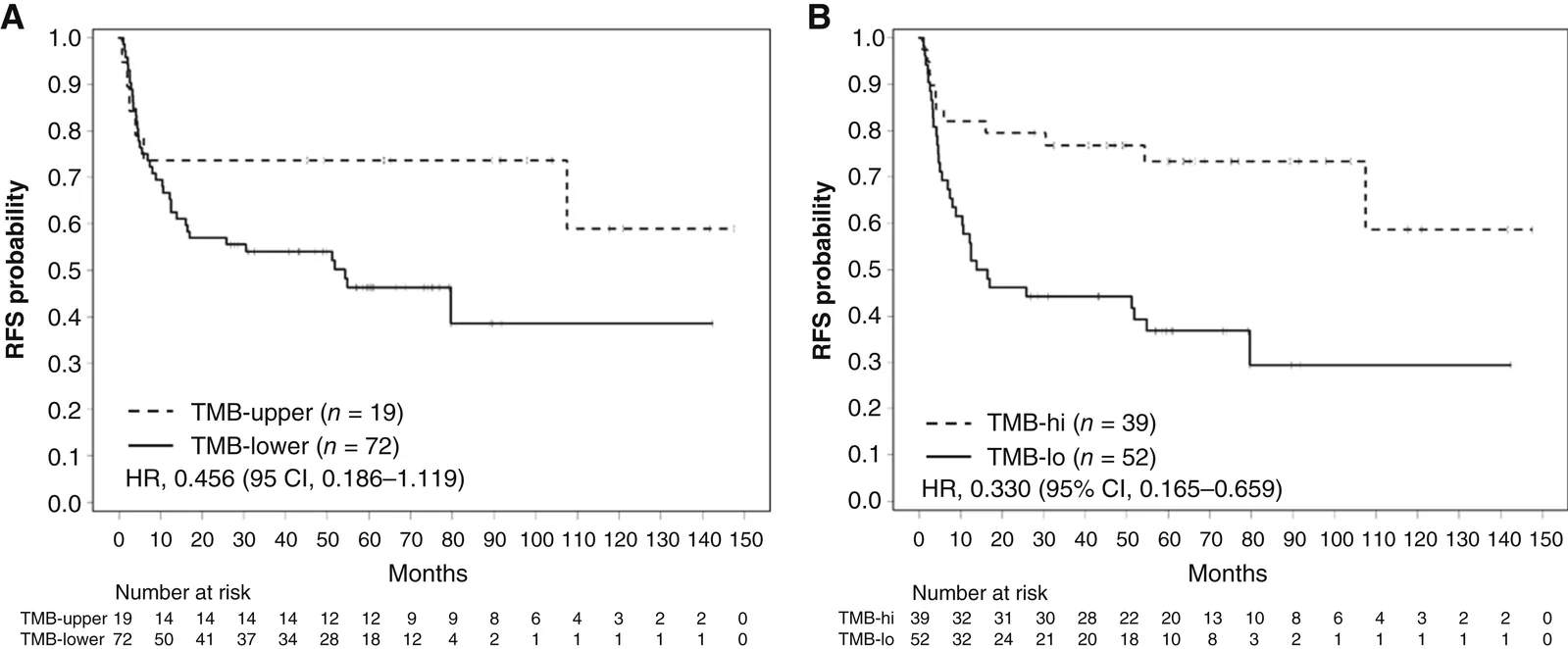
RFS by TMB. **A,** Upper TMB quintile (TMB-upper) vs. lower four TMB quintiles (TMB-lower). **B,** TMB ≥ 10 Mut/Mb (TMB-hi) vs. TMB < 10 Mut/Mb (TMB-lo). CI, confidence interval.

We next determined the impact of TMB levels on relapse rates and RFS. The median TMB for relapsed and nonrelapsed patients was 7.7 and 10.5, respectively. Relapse rates by TMB threshold are shown in Table 1. For patients in the upper TMB quintile, median RFS was not reached versus 54.2 months in the lower four quintiles [HR, 0.456 (0.186–1.119); *P* = 0.08; Fig. 2A]. For patients with TMB of 10 or greater, median RFS was not reached versus 15.1 months for TMB less than 10 [HR, 0.330 (0.165–0.659); *P* < 0.001; Fig. 2B].

**Table 1.** Relapse rate by TMB.

TMB group	Relapse status, *n* (%)		OR
	Yes	No	
Lower quintiles	34 (47.2%)	38 (52.8%)	0.30, *P* = 0.065
Upper quintile	4 (21.1%)	15 (79%)	​
Lower tertile	19 (65.5%)	10 (34.5%)	0.23, *P* = 0.006
Middle tertile	11 (35.5%)	20 (64.5%)	​
Upper tertile	8 (25.8%)	23 (74.2%)	​
Less than 10 (Mut/Mb)	29 (55.8%)	23 (44.2%)	0.24, *P* = 0.003
10 (Mut/Mb) or higher	9 (23.1%)	30 (76.9%)	​

**Figure 3.**
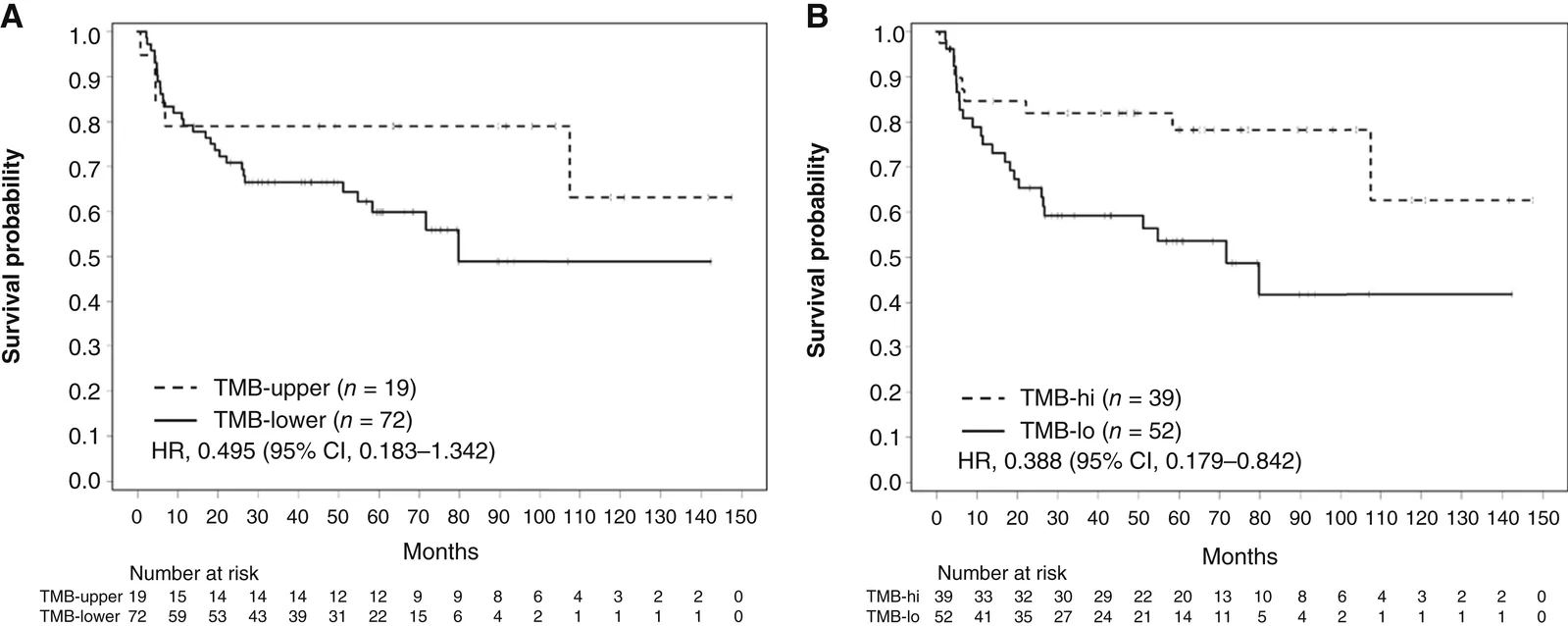
OS by TMB. **A,** Upper TMB quintile (TMB-upper) vs. lower four TMB quintiles (TMB-lower). **B,** TMB ≥ 10 Mut/Mb (TMB-hi) vs. TMB < 10 Mut/Mb (TMB-lo). CI, confidence interval.

We next assessed the impact of TMB rates on OS. For patients in the upper TMB quintile, median OS was not reached versus 79.8 months in the lower four TMB quintiles [HR, 0.495 (0.183–1.342); *P* = 0.16; Fig. 3A]. For patients with TMB of 10 or greater, median OS was not reached versus 71.7 months for TMB less than 10 [HR, 0.388 (0.179–0.842); *P* = 0.013; Fig. 3B].

### Gene variants and differential gene expression associated with high TMB

After characterizing the relationship between TMB and clinical outcomes in this cohort, we performed exploratory analyses to identify genomic variants and gene expression patterns to gain insight into the molecular landscape associated with high TMB in MIBC. We assessed whether tumors in the upper TMB quintile were enriched for pathogenic mutations in genes associated with high TMB, such as DNA polymerases (*POLE*, *POLD1*) and mismatch repair (MMR; ref. 24). No mutations in *POLE* or *POLD1* genes were detected in this cohort. Nine patients had pathogenic MMR mutations [*MLH1* (*n* = 2), *MLH3* (*n* = 1), *MSH2* (*n* = 1), *MSH3* (*n* = 1), *MSH6* (*n* = 2), and *PMS2* (*n* = 2)]. Both tumors with *MSH6* mutations (mean TMB 17.1) were in the upper TMB quintile. In aggregate, the presence of an MMR mutation trended toward enrichment in the highest TMB quintile (OR = 3.57; *P* = 0.087) but was not significantly associated with achieving pCR (OR = 0.58; *P* = 0.713). Among 23 genes with pathogenic mutations detected in at least five patients in the cohort, only mutations in the *EP300* gene (*n* = 7) were significantly enriched in the upper TMB quintile (OR = 12.50; *P* = 0.004) although, interestingly, none of the tumors with *EP300* mutations achieved pCR.

Sixty-one (67%) patients underwent RNA expression profiling. Of these, 13 (21.3%) samples had TMB values in the upper quintile. We performed supervised clustering to identify differentially expressed genes (DEG) between the high (upper quintile) and nonhigh (lower four quintiles) TMB groups. Forty-four genes demonstrated statistically significant differences in expression between the TMB high and nonhigh groups when adjusted for multiplicity (Supplementary Table S2). Kyoto Encyclopedia of Genes and Genomes pathway analysis was also performed on DEGs and suggested that the high TMB environment may be enriched for dysregulated glycosphingolipid (GSL) production, but the cohort size precludes firm conclusions (Supplementary Table S3).

## Discussion

In this study, we evaluated the prognostic value of TMB in MIBC based on the hypothesis that high TMB would identify patients unlikely to benefit from NAC. However, we observed the opposite association. Although analyses using the upper TMB quintile and tertile demonstrated consistent numerical trends toward improved pCR rates and survival, statistically significant associations with pathologic response, RFS, and OS were observed at the established threshold of TMB ≥ 10 Mut/Mb.

Because clinically validated TMB thresholds have not been established in MIBC, we defined high TMB as the upper quintile of the cohort (TMB ≥ 14.7 Mut/Mb). In the upper TMB quintile, strong trends toward improved pCR (OR, 0.43; *P* = 0.165), RFS (HR, 0.456; *P* = 0.08), and OS (HR, 0.495; *P* = 0.16) were observed. This finding is particularly striking as the study was powered to detect the opposite association between TMB and clinical outcomes. We also evaluated the TMB cutoff ≥ 10 Mut/Mb, which has been validated as predictive of ICI benefit across tumor types. Patients with TMB ≥ 10 Mut/Mb were more likely to achieve pCR (OR, 0.39; *P* = 0.044) and demonstrated significantly improved RFS (HR, 0.330; *P* < 0.001) and OS (HR, 0.388; *P* = 0.013).

To our knowledge, this is the first study to demonstrate that TMB ≥ 10 Mut/Mb is prognostic in patients with MIBC treated with NAC independent of ICI exposure. Prior studies have identified individual genomic alterations associated with improved pCR rates in MIBC; however, our findings may represent the first biomarker to be associated with improvements in both RFS and OS in patients receiving NAC. Importantly, the very low use of adjuvant ICIs in this cohort (4.4%) minimizes confounding from immunotherapy, which has become a standard component of contemporary perioperative regimens.

In contrast to our findings in localized disease, TMB was not associated with improved response rates in patients with advanced urothelial carcinoma treated with chemotherapy alone or in combination with pembrolizumab on KEYNOTE-361 in the metastatic setting (25). This discrepancy may reflect fundamental biological differences between localized and metastatic disease. Specifically, the substantially higher tumor cell burden in overt metastases may increase the likelihood of chemotherapy-resistant subclones, driven by underlying genomic heterogeneity and mutational evolution.

Since the initiation of this study, the standard of care for MIBC has evolved beyond neoadjuvant cisplatin-based chemotherapy alone and now routinely includes the use of perioperative ICIs (1, 2). Although few patients in our cohort received ICIs, our findings suggest that the prognostic value of TMB may be even greater in contemporary regimens that combine chemotherapy with immunotherapy. This hypothesis is supported by KEYNOTE-361, in which high TMB was positively associated with PFS and OS in the metastatic setting among patients receiving combined chemotherapy and pembrolizumab compared with chemotherapy alone (25). Together, these data support further investigation of TMB as a prognostic biomarker in perioperative chemoimmunotherapy for MIBC.

Emerging systemic options underscore the relevance of TMB as a biomarker in MIBC. The combination of the antibody–drug conjugate enfortumab vedotin (EV), which delivers the cytotoxic payload monomethyl auristatin E, with pembrolizumab has demonstrated robust activity in MIBC and is increasingly being adopted as a cisplatin-sparing perioperative option as regulatory approvals expand (26). In the metastatic setting, real-world analyses of EV monotherapy identified high TMB as a potential prognostic factor for OS, with improved median OS observed in TMB-high compared with non–TMB-high patients (13.6 vs. 8.3 months; *P* = 0.014; ref. 27). These findings warrant evaluation of whether TMB similarly stratifies outcomes in patients with MIBC treated with perioperative EV-based regimens.

Using gene variant and expression data, we examined the molecular landscape associated with the upper TMB quintile to gain potential mechanistic insight into the high TMB setting in MIBC. Tumors with deficient DNA MMR are strongly associated with high TMB (28). Consistent with this biology, patients harboring pathogenic MMR gene alterations in our cohort trended toward enrichment in the upper TMB quintile (OR, 3.57; *P* = 0.087). Pathogenic mutations in *EP300* were significantly associated with the upper TMB quintile (OR = 12.50; *P* = 0.004). Interestingly, neither MMR gene alterations nor *EP300* mutations were clearly associated with an increased likelihood of achieving pCR, suggesting that the underlying genomic mechanisms driving the favorable outcomes in the highest TMB quintile are multifactorial. The observed enrichment of MMR gene alterations in the upper TMB quintile raises the possibility that mutations in additional DNA damage response (DDR) genes might confound the association between TMB and outcomes. However, no other DDR gene alterations were enriched in the upper TMB quintile. Furthermore, no *ERCC2* gene mutations were detected in this cohort, arguing against significant DDR-mediated confounding of the observed findings. Importantly, this study was not adequately powered to detect definitive associations between gene variants and the upper TMB quintile, so these findings should be considered exploratory and hypothesis-generating.

Additional mechanisms may account for the favorable association observed between high TMB and clinical outcomes in this cohort. Higher mutational burden may be associated with increased neoantigen load and a more immunogenic tumor microenvironment, which could contribute to enhanced immune-mediated tumor cell death as an indirect effect of cytotoxic therapy although this finding has not been consistently observed (29, 30).

Interestingly, the subset of patients who underwent RNA analysis demonstrated potential dysregulation of GSL biosynthesis in the high TMB group. GSLs are involved in immune signaling and modulation, raising the possibility that altered GSL production may influence susceptibility to chemotherapy-induced, immune-mediated cell death (31, 32). However, this observation should be considered exploratory given the limited sample size and lack of validation although it provides potential mechanistic insight that warrants further study.

Several limitations should be acknowledged. This study analyzed a cohort treated in an era in which patients predominantly received neoadjuvant cisplatin-based chemotherapy without perioperative immunotherapy. Although treatment paradigms are evolving, cisplatin-based chemotherapy remains widely utilized globally, and these findings provide biological insights and generate hypotheses that may ultimately inform patient selection across current and emerging therapeutic strategies.

Although the cohort size was modest, the sample size was prespecified, clinical outcomes were mature and consistent with historical controls, and significant associations were observed despite limited power, supporting the robustness of the findings. The cohort size was insufficient to support robust multivariable analyses of baseline clinical factors. Notably, all four patients who received adjuvant immune checkpoint inhibition were within the lower four TMB quintiles (Supplementary Table S1); however, given the low overall utilization, this is unlikely to have meaningfully confounded RFS outcomes. We also observed an imbalance in chemotherapy regimens between TMB groups, with a higher proportion of patients in the upper quintile receiving MVAC-based therapy. Although prior studies have suggested that neoadjuvant MVAC-based therapy may offer improved efficacy compared with G/C, this has not been consistently observed and was not supported by univariable analysis of relapse rates in our cohort (33, 34).

An important strength of this study is the use of a standardized, commercially available genomic assay, enhancing the generalizability of the results and avoiding limitations inherent to institution-specific sequencing platforms and bioinformatics pipelines.

In summary, we demonstrated that TMB ≥ 10 Mut/Mb was associated with improved pathologic response, RFS, and OS in this cohort of patients with MIBC treated with NAC. These findings provide a strong rationale for the prospective evaluation of TMB as a biomarker in contemporary perioperative regimens incorporating immunotherapy and novel systemic agents.

## Supplementary Material

Supplementary Figure S1 Figure S1. Survival outcomes for entire study cohort.

Supplementary Table S1 Table S1. Patient Characteristics

Supplementary Table S2 Table S2. Differentially expressed genes between the upper and lower four TMB quintiles.

Supplementary Table S3 Table S3. KEGG Pathway Analysis

## Data Availability

The genomic data generated and analyzed in this study were derived from a commercial sequencing platform (Tempus AI, Inc.) and are subject to data use agreements that restrict public deposition of raw sequencing data. Deidentified data supporting the findings of this study may be made available from the corresponding author upon reasonable request, subject to institutional approvals and applicable data sharing agreements.
