# Supplementary material for: Tumor Mutational Burden and Clinical Outcomes in Patients with Muscle-Invasive Bladder Cancer Treated with Neoadjuvant Chemotherapy
Source: Cancer Res Commun. 2026 Aug 27;6(8):2010–6. doi: 10.1158/2767-9764.CRC-26-0255 (PMC13519591; doi:10.1158/2767-9764.CRC-26-0255)
Supplement: Supplementary file 1 [file crc-26-0255_supplementary_table_s1_suppst1.docx]

|  | |  | **n (%)** | **n (%)** | **n (%)** |
| --- | --- | --- | --- | --- | --- |
|  |  |  | **Total (N = 91)** | **TMB upper** (N = 19)** | **TMB lower*** (N = 72)** |
| *Gender* | |  |  |  |  |
|  | Male | | 63 (69) | 13 (68) | 50 (69) |
|  | Female | | 28 (31) | 6 (32) | 22 (31) |
| *Race* | |  |  |  |  |
|  | Caucasian | | 80 (88) | 15 (79) | 65 (90) |
|  | Black | | 10 (11) | 4 (21) | 6 (8) |
|  | Other | | 1 (1) | 0 (0) | 1 (1) |
| *Chemotherapy* | |  |  |  |  |
|  | Gemcitabine/Cisplatin | | 59 (65) | 7 (37) | 52 (72) |
|  | MVAC or ddMVAC* | | 32 (35) | 12 (63) | 20 (28) |
| *Adjuvant Immunotherapy* | | |  |  |  |
|  | Yes |  | 4 (4) | 0 (0) | 4 (6) |
|  | No |  | 87 (96) | 19 (100) | 68 (94) |
| *Pathologic Stage* | | |  |  |  |
|  | 0 |  | 29 (32) | 9 (47) | 20 (28) |
|  | 0A |  | 1 (1) | 0 (0) | 1 (1) |
|  | I |  | 9 (10) | 2 (11) | 7 (10) |
|  | II |  | 13 (14) | 2 (11) | 11 (15) |
|  | IIIA |  | 22 (24) | 3 (16) | 19 (26) |
|  | IIIB |  | 16 (18) | 2 (11) | 14 (19) |
|  | IVA |  | 1 (1) | 1 (5) | 0 (0) |
| * (dose dense) methotrexate, vinblastine, doxorubicin, cisplatin | | | |  |  |
| ** upper quintile | |  |  |  |  |
| *** lower four quintiles | | |  |  |  |

Supplemental Table 1. Patient Characteristics
