# Supplementary material for: Tumor Mutational Burden and Clinical Outcomes in Patients with Muscle-Invasive Bladder Cancer Treated with Neoadjuvant Chemotherapy
Source: Cancer Res Commun. 2026 Aug 27;6(8):2010–6. doi: 10.1158/2767-9764.CRC-26-0255 (PMC13519591; doi:10.1158/2767-9764.CRC-26-0255)
Supplement: Supplementary file 2 [file crc-26-0255_supplementary_table_s2_suppst2.docx]

Supplemental Table 2. Differentially expressed genes between the upper and lower four TMB quintiles.

| ensembl | hgnc_symbol | log2FoldChange | pvalue | padj |
| --- | --- | --- | --- | --- |
| ENSG00000053438 | NNAT | 6.068575 | 1.70E-05 | 7.31E-03 |
| ENSG00000253998 | IGKV2-29 | 3.116237 | 2.47E-04 | 3.69E-02 |
| ENSG00000222036 | POTEM | 2.444503 | 6.22E-04 | 5.90E-02 |
| ENSG00000187537 | POTEG | 2.404584 | 1.01E-03 | 7.55E-02 |
| ENSG00000225308 | ASS1P11 | 2.288856 | 1.59E-03 | 9.22E-02 |
| ENSG00000167414 | GNG8 | 2.034717 | 1.80E-04 | 3.16E-02 |
| ENSG00000182902 | SLC25A18 | 1.681055 | 1.11E-04 | 2.48E-02 |
| ENSG00000119457 | SLC46A2 | 1.609877 | 1.01E-05 | 4.99E-03 |
| ENSG00000211973 | IGHV1-69 | 1.508784 | 1.38E-03 | 8.59E-02 |
| ENSG00000183778 | B3GALT5 | 1.448143 | 9.27E-04 | 7.35E-02 |
| ENSG00000152192 | POU4F1 | 1.424748 | 7.89E-04 | 6.83E-02 |
| ENSG00000186564 | FOXD2 | 1.355007 | 1.31E-04 | 2.69E-02 |
| ENSG00000124215 | CDH26 | 1.31268 | 2.50E-04 | 3.71E-02 |
| ENSG00000149403 | GRIK4 | 1.301011 | 4.35E-04 | 5.01E-02 |
| ENSG00000175591 | P2RY2 | 1.293102 | 2.54E-05 | 9.47E-03 |
| ENSG00000196549 | MME | 1.17271 | 4.37E-04 | 5.01E-02 |
| ENSG00000198062 | POTEH | 1.126913 | 1.38E-03 | 8.60E-02 |
| ENSG00000167964 | RAB26 | 1.076945 | 5.97E-04 | 5.84E-02 |
| ENSG00000065325 | GLP2R | -1.047958 | 1.79E-03 | 9.95E-02 |
| ENSG00000146250 | PRSS35 | -1.095796 | 1.42E-03 | 8.74E-02 |
| ENSG00000144218 | AFF3 | -1.104391 | 6.38E-04 | 5.95E-02 |
| ENSG00000178187 | ZNF454 | -1.1097 | 4.67E-04 | 5.15E-02 |
| ENSG00000162267 | ITIH3 | -1.19117 | 5.30E-04 | 5.48E-02 |
| ENSG00000133937 | GSC | -1.208373 | 1.56E-03 | 9.22E-02 |
| ENSG00000174460 | ZCCHC12 | -1.217301 | 7.06E-04 | 6.34E-02 |
| ENSG00000147606 | SLC26A7 | -1.387611 | 1.17E-03 | 8.01E-02 |
| ENSG00000105642 | KCNN1 | -1.473341 | 1.90E-04 | 3.16E-02 |
| ENSG00000122756 | CNTFR | -1.499695 | 3.75E-04 | 4.88E-02 |
| ENSG00000185070 | FLRT2 | -1.535849 | 1.27E-03 | 8.30E-02 |
| ENSG00000166106 | ADAMTS15 | -1.541158 | 4.46E-05 | 1.40E-02 |
| ENSG00000169994 | MYO7B | -1.56843 | 1.42E-04 | 2.83E-02 |
| ENSG00000147488 | ST18 | -1.647799 | 7.91E-05 | 2.02E-02 |
| ENSG00000041515 | MYO16 | -1.725809 | 2.84E-05 | 1.01E-02 |
| ENSG00000159337 | PLA2G4D | -1.813652 | 2.20E-04 | 3.39E-02 |
| ENSG00000132321 | IQCA1 | -1.894731 | 1.98E-05 | 8.17E-03 |
| ENSG00000112936 | C7 | -2.018359 | 1.64E-04 | 3.08E-02 |
| ENSG00000164764 | SBSPON | -2.027797 | 1.13E-05 | 5.30E-03 |
| ENSG00000100433 | KCNK10 | -2.207605 | 3.23E-05 | 1.10E-02 |
| ENSG00000130876 | SLC7A10 | -2.323326 | 1.01E-04 | 2.36E-02 |
| ENSG00000196136 | SERPINA3 | -2.584925 | 2.89E-06 | 2.20E-03 |
| ENSG00000182585 | EPGN | -2.685582 | 5.39E-04 | 5.52E-02 |
| ENSG00000100593 | ISM2 | -2.738537 | 1.92E-04 | 3.16E-02 |
| ENSG00000109205 | ODAM | -3.027421 | 8.91E-05 | 2.17E-02 |
| ENSG00000139219 | COL2A1 | -5.374816 | 2.52E-09 | 6.22E-06 |
