# Supplementary material for: Tumor Mutational Burden and Clinical Outcomes in Patients with Muscle-Invasive Bladder Cancer Treated with Neoadjuvant Chemotherapy
Source: Cancer Res Commun. 2026 Aug 27;6(8):2010–6. doi: 10.1158/2767-9764.CRC-26-0255 (PMC13519591; doi:10.1158/2767-9764.CRC-26-0255)
Supplement: Supplementary file 3 [file crc-26-0255_supplementary_table_s3_suppst3.docx]

Supplemental Table 3. KEGG Pathway Analysis

| Term | P.value | Adjusted.P.value | Odds.Ratio | Combined.Score | Genes |
| --- | --- | --- | --- | --- | --- |
| glycosphingolipid biosynthesis lactoseries | 0.009462 | 0.036791 | 123.28395 | 574.56772 | B3GALT5 |
| glycosphingolipid biosynthesis globoseries | 0.013222 | 0.036791 | 85.333333 | 369.13902 | B3GALT5 |
| renin angiotensin system | 0.016034 | 0.036791 | 69.322917 | 286.51434 | MME |
| neuroactive ligand receptor interaction | 0.021106 | 0.036791 | 9.842971 | 37.97592 | P2RY2;GRIK4 |
| alzheimers disease | 0.026279 | 0.036791 | 41.057613 | 149.40806 | MME |
| glycan structures biosynthesis 2 | 0.055511 | 0.064763 | 18.758945 | 54.23541 | B3GALT5 |
| hematopoietic cell lineage | 0.080405 | 0.080405 | 12.703704 | 32.02195 | MME |
